# An international, cross-sectional survey of preprint attitudes among biomedical researchers

**DOI:** 10.12688/f1000research.143013.1

**Published:** 2024-01-03

**Authors:** Jeremy Y. Ng, Valerie Chow, Lucas J. Santoro, Anna Catharina Vieira Armond, Sanam Ebrahimzadeh Pirshahid, Kelly D. Cobey, David Moher

**Affiliations:** 1 Centre for Journalology, Ottawa Methods Centre, Ottawa Hospital Research Institute, Ottawa, Ontario, K1H 8L6, Canada; 2 Department of Health Research Methods, Evidence, and Impact, Faculty of Health Sciences, McMaster University, Hamilton, Ontario, L8S 4L8, Canada; 3 School of Epidemiology and Public Health, Faculty of Medicine, University of Ottawa, Ottawa, Ontario, K1G 5Z3, Canada; 4 Meta-Research and Open Science Program, University of Ottawa Heart Institute, Ottawa, Ontario, K1Y 4W7, Canada

**Keywords:** biomedicine, open science, open science practices, preprinting, preprints, researchers

## Abstract

**Background:**

Preprints are scientific manuscripts that are made available on open-access servers but are not yet peer reviewed. Although preprints are becoming more prevalent, uptake is not uniform or optimal. Understanding researchers’ opinions and attitudes toward preprints is valuable for their successful implementation. Understanding knowledge gaps and researchers’ attitudes toward preprinting can assist stakeholders, such as journals, funding agencies, and universities, to implement preprints more effectively. Here, we aimed to collect perceptions and behaviours regarding preprints across an international sample of biomedical researchers.

**Methods:**

Biomedical authors were identified by a keyword-based, systematic search of the MEDLINE database, and their emails were extracted to invite them to our survey. A cross-sectional anonymous survey was distributed to all identified biomedical authors to collect their knowledge, attitudes, and opinions regarding preprinting.

**Results:**

The survey was completed by 730 biomedical researchers with a response rate of 3.20% and demonstrated a wide range of attitudes and opinions about preprints with authors from various disciplines and career stages worldwide. Most respondents were familiar with the concept of preprints but most had not previously published a preprint. The lead author of the project and journal policy had the greatest impact on decisions to post a preprint, whereas employers/research institutes had the least impact. Supporting open science practices was the highest ranked incentive, while increasing authors’ visibility was the highest ranked motivation for publishing preprints.

**Conclusions:**

Although many biomedical researchers recognize the benefits of preprints, there is still hesitation among others to engage in this practice. This may be due to the general lack of peer review of preprints and little enthusiasm from external organizations such as journals, funding agencies, and universities. Future work is needed to determine optimal ways to increase researchers’ attitudes through modifications to current preprint systems and policies.

**List of abbreviations**
CHERRIES: Checklist for Reporting Results of Internet E-Surveys
FAST: Focused, Appropriate, Specific and Transparent
OSF: Open Science Framework

## Introduction

The term “preprints” refers to scientific manuscripts that are made available on an open-access infrastructure, known as the “preprint server,” which has not yet undergone formal peer review ^1^ ( https://asapbio.org/). One of the primary purposes of preprints is to make research available as quickly as possible, given the time lag from journal submission to publication, which typically takes around 8 to 10 months in biomedical sciences due to multiple review rounds, editorial decision-making, high rejection rates, and time needed for revisions. ^1^^–^^5^ Preprints do not rely on peer review prior to release, allowing knowledge to be shared quickly with the introduction of novel results and methodology that can save months to years of research time. ^4^ Preprints have become incredibly valuable for authors, as a larger group of researchers can critique their work (i.e., open peer review) with the preprint servers’ feedback system that allows for public and open feedback directly onto a manuscript that encourages discussion ( https://asapbio.org/). The focused, appropriate, specific, and transparent (FAST) principles, which are guidelines that reviewers can follow when reviewing preprints, allow for high-quality, constructive feedback to be provided. ^6^ As a result, researchers can improve their final manuscript, so it can possibly be published sooner and with fewer revisions. ^2^ In addition, authors have also begun to cite preprints more frequently in their working manuscripts as institutions and funders have become more open and permissible in their policies regarding preprints. ^2^ Furthermore, funders acknowledge the importance of preprints by encouraging researchers to post and reference preprints in their grant applications. ^2^ Preprint servers still utilize a minimal screening process to evaluate articles for incompleteness, plagiarism, and whether they blatantly disregard or contradict widely accepted medical practices that could potentially jeopardize someone’s health if posted. ^7^

However, despite their benefits, many scientists are hesitant about preprinting. Major concerns include the credibility of preprints and premature media coverage. ^2^^,^^5^^,^^8^ During the COVID-19 pandemic, online media used preprints to rapidly introduce novel research to the public. However, patients and the public may be less familiar with the fact that preprints have not yet undergone the peer review process and may fail to discern the difference between a preprint and an article published in peer reviewed literature. ^2^^,^^8^ Nonetheless, much COVID-19 research posted on preprint servers has received high levels of coverage, and certain preprints could be used to push certain agendas, such as conspiracy theories and xenophobia. ^8^ This has led to the spread of misinformation. ^8^^,^^9^

The purpose of this study was to explore biomedical researchers’ attitudes toward preprinting through the administration of an anonymous cross-sectional online survey. We aimed to determine the factors that influence biomedical researchers’ opinions about publishing and viewing preprints. Thus, future work should focus on determining how to improve researchers’ attitudes toward preprinting. Additionally, our results may potentially impact how other stakeholders implement and modify their preprint policies in the future.

## Methods

### Approach

Prior to commencing this study, a protocol was registered in the Open Science Framework ( OSF) before participant recruiting began. ^10^ The MEDLINE database was used to identify participants using publicly available information. Once identified, the participants were invited to complete an online survey. Survey questions were purpose-built by a core team of authors. They focused on understanding biomedical researchers’ attitudes and opinions concerning preprinting. This study was approved by the Ottawa Health Sciences Research Ethics Board (OHSN-REB Number: 20220584-01H) on November 17, 2022.

### Open science statement

The protocol, study materials, and raw and analyzed data have been made available via OSF at the time of publication submission. ^10^ A preprint of our manuscript has been posted on MedRxiv.

### Study design

We conducted an anonymous, online, cross-sectional, closed survey of authors published in biomedical journals.

### Sampling framework

The sampling method followed that described by Ebrahimzadeh *et al*. ^11^ We downloaded the MEDLINE database that contained approximately 30,000 journals. From this list, 400 journals were randomly selected using the RAND() function of Microsoft Excel for Microsoft 365 MSO (Version 2310 Build 16.0.16924.20054) (2021). Authors’ names and emails were extracted using our script from articles published from 2021/07/01 to 2022/08/01. Extended data: Supplementary File 1 outlines the semi-automated process we used, generated by Ebrahimzadeh *et al*. ^11^^,^^12^

### Participant recruitment

Only researchers who were identified by our sampling framework developed by Ebrahimzadeh *et al*. ^11^ received a closed survey. Using SurveyMonkey, the prospective participants received an email invitation to complete the survey. The email included an authorized recruitment script that described the study and its goals, invited recipients to review an informed consent form, and asked them to complete an anonymous online survey. Upon clicking on the survey link in the invitation email, participants had to read and agree with the informed consent form before being able to see the survey. The initial list of 24 000 names and e-mails of the corresponding authors contained duplicates and no longer functioning e-mails. We were able to determine that functioning emails were no longer available due to SurveyMonkey’s bounced function, which labels certain contact information that has a permanent reason for an email or text to not be delivered. Before recruitment, duplicate authors and no longer functioning emails were deleted from the dataset. Therefore, 22 808 corresponding authors were emailed. We predicted that we would receive approximately 2200 responses based on a predicted response rate of approximately 10%. There was no financial compensation or requirements for participation. Anyone who did not want to respond to a question could skip it.

### Survey

The complete survey is provided in the Extended data: Supplementary File 2. ^12^ The survey was created, pilot tested by SEP, DM, KC, and ACA, distributed, and collected using the University of Ottawa’s SurveyMonkey account. The survey contained 29 questions, and SurveyMonkey estimated that it would take approximately 15 minutes to complete. The survey initially asked participants five general demographic questions including gender, research role, area of research, and country of residence. They were then asked nine questions about their experiences with preprints and fifteen questions about their preferences and opinions regarding preprinting. The survey used adaptive formatting, which means that each participant’s prior response influences the next question they see and the answers that might be provided. Most of the questions were multiple-choice; the remaining questions were open-ended and required participants to write their responses in a text box. Reminder emails were sent to the participants after the first and second weeks following the original invitation. The survey was closed approximately five weeks after the initial invitation email was sent. Both the initial invitation and reminder emails made this clear to the survey takers. Responses were collected from January to February 2023.

### Data management and analysis

In accordance with Ebrahimzadeh *et al*., ^11^ survey data gathered from the participants were exported to Microsoft Excel. Basic descriptive statistics, such as counts and percentages, were generated based on the analysis of the quantitative data. Based on gender, employment sector, current career stage, and research area, information and replies from participants were compared and studied. Thematic content analysis was used by different members of the research team to individually code replies to specific qualitative items. To reach an agreement on the respective codes, which are categorically classified and specified into distinct tables, researchers carried out multiple rounds of discussions. The Checklist for Reporting Results of Internet E-Surveys (CHERRIES) was used to report this survey. ^13^

## Results

### Demographics

Our survey received 730 responses, with a response rate of 3.20% and a completion rate of 95.48%. Incomplete responses were defined as those with no questions answered on the second page of the survey. Moreover, we have reported our raw response rate, which is underestimated because we cannot determine how many of the 22 808 authors who were emailed identified as biomedical researchers or had an actively working email address. All questions were optional; therefore, we provided the total number of respondents for each question. The percentages provided were appropriately calculated using each question’s total number of responses. Over three-fifths of respondents (n=455, 62.41%) were identified as senior researchers, which we defined as researchers who started their careers after formal education over ten years ago. Of the 729 responses, participants were identified as follows: academia (n=580, 79.56%), research staff with no formal academic or industry position (n=58, 7.96%), government scientists (n=28, 3.84%), third sector (n=8, 1.10%), pharmaceutical industry (n=5, 0.69%), and none of the participants were part of the scholarly communication industry. We received 50 responses (6.86%) that chose the “other” option, which mainly consisted of participants from the biotechnology industry, retired personnel, clinical researchers, clinicians, medical practitioners, and students. Detailed demographics and other aggregate participant data are presented in Table 1. The responses from participants were also categorized based on gender, employment sector, current career stage, and research area.

**Table 1.** Characteristics of survey respondents.

Gender (n=728)	
Male	401 (55.08%)
Female	312 (42.86%)
Prefer not to indicate	14 (1.92%)
Non-binary	1 (0.14%)
Country of primary affiliation (n=698)	
United States of America	189 (27.08%)
Canada	69 (9.89%)
India	41 (5.87%)
United Kingdom of Great Britain and Northern Ireland	38 (5.44%)
Australia	34 (4.87%)
Spain	32 (4.58%)
China	29 (4.15%)
Italy	25 (3.58%)
Brazil	18 (2.58%)
Netherlands	18 (2.58%)
Iran (Islamic Republic of)	16 (2.29%)
Turkey	14 (2.01%)
Germany	12 (1.72%)
Switzerland	12 (1.72%)
France	11 (1.58%)
Denmark	9 (1.29%)
Greece	9 (1.29%)
Mexico	8 (1.15%)
Japan	7 (1.00%)
Norway	7 (1.00%)
Austria	6 (0.86%)
Republic of Korea	6 (0.86%)
Belgium	5 (0.72%)
Colombia	5 (0.72%)
Poland	5 (0.72%)
Romania	5 (0.72%)
Sweden	5 (0.72%)
Indonesia	4 (0.57%)
Portugal	4 (0.57%)
Russian Federation	4 (0.57%)
Thailand	4 (0.57%)
Chile	3 (0.43%)
Ireland	3 (0.43%)
Pakistan	3 (0.43%)
United Arab Emirates	3 (0.43%)
Albania	2 (0.29%)
Bangladesh	2 (0.29%)
Czech Republic	2 (0.29%)
Jordan	2 (0.29%)
Malaysia	2 (0.29%)
New Zealand	2 (0.29%)
Other *	17 (2.38%)
Current career stage (n=729)	
Senior researcher	455 (62.41%)
Mid-career researcher	139 (19.07%)
Early career researcher	89 (12.21%)
Other (please specify)	25 (3.43%)
Graduate student	21 (2.88%)
Current employment sector (n=729)	
Academic	580 (79.56%)
Research staff (no formal academic/industry position)	58 (7.96%)
Other (please specify)	50 (6.86%)
Government scientist	28 (3.84%)
Third sector (e.g., NGO, non-profit)	8 (1.10%)
Pharmaceutical industry	5 (0.69%)
Current research area (n=727)	
Clinical research	335 (46.08%)
Other (please specify)	125 (17.19%)
Pre-clinical research - in vivo	77 (10.59%)
Health systems research	74 (10.18%)
Pre-clinical research - in vitro	72 (9.90%)
Methods research	44 (6.05%)

* Total number of countries with one response.

### Experience

*Familiarity with preprints*

We asked how familiar the participants were with the concept of a preprint based on the given definition, “A publicly available version of any type of scientific manuscript/research output preceding formal publication” ( https://forrt.org/). Of the 694 responses, participants ranked how familiar they were with the concept of preprints: very familiar (n=167, 24.06%), familiar (n=269, 38.76%), and somewhat familiar (n=92, 13.26%). Only 61 (8.79%) respondents had never heard of this term.

*Publishing experience*

We then asked about the participants’ personal experiences with posting on preprint servers. Of the 694 responses, approximately one-third (n=237, 34.15%) had authored more than 31 publications in the past five years. Most participants had not posted a manuscript on a preprint server, with 540 (78.15%) participants of 691 responses not posting their most recent work on a preprint server, and 414 (59.74%) respondents of 693 responses having never posted a manuscript on a preprint server. However, when asked if participants would create a preprint in the future, we received 695 responses: 303 (43.60%) participants said they would not, 296 (42.59%) participants said they were unsure if they would, and 96 (13.81%) participants said they would create a preprint in the future.

Of the 335 respondents who had posted preprints before, the most common preprint server used was bioRxiv, with 148 (44.18%) respondents having published previously on this server. In addition, we asked at what point in the publication process were preprints posted, and of the 294 respondents who had previously posted preprints, 110 (37.41%) participants submitted their manuscripts prior to submitting them to a journal, while 133 (45.24%) participants posted a preprint simultaneously while submitting to a journal.

*Viewing/downloading preprints*

Interestingly, although most participants did not publish their own works on preprint servers, more than two-thirds of the 695 respondents (n=481, 69.21%) had previously viewed or downloaded preprints, although only approximately a quarter (n=176, 25.32%) had cited a preprint. The most common preprint servers used to view/download preprints among 682 responses were bioRxiv (n=243, 35.63%), MedRxiv (n=148, 21.70%), and ResearchGate (n=140, 20.53%).

*Peer reviewing preprints*

We asked whether peer review should become a part of the preprinting process, and 684 responded, with 231 (33.77%) respondents believing that peer review should be part of the preprinting process, 240 (35.09%) respondents said it should not, while 213 respondents (31.14%) were unsure ( Table 2). We also asked if our participants had experience with peer reviewing a preprint, and from the 688 responses, the vast majority (n=638, 92.73%) stated they had not. Of those who had peer reviewed a preprint, we wanted to know if the FAST principles were used during the process, and we received 205 responses. Of the respondents, 148 (72.20%) stated that they were not familiar with the FAST principles, 44 (21.46%) said they did not use the FAST principles, and 13 (6.34%) said they did.

**Table 2.** Respondent’s opinion on mandating peer review in the preprinting process (n=684).

Yes	231 (33.77%)
No	240 (35.09%)
I am not sure	213 (31.14%)

We also asked if participants believed that patients or members of the public should be able to peer review preprints. Of the 445 who expressed an opinion, 320 (71.91%) believed that patients and members of the public should not be able to peer review preprints, while 125 (28.09%) believed that they should be able to.

### Factors and attitudes toward preprinting

**Figure 1.**
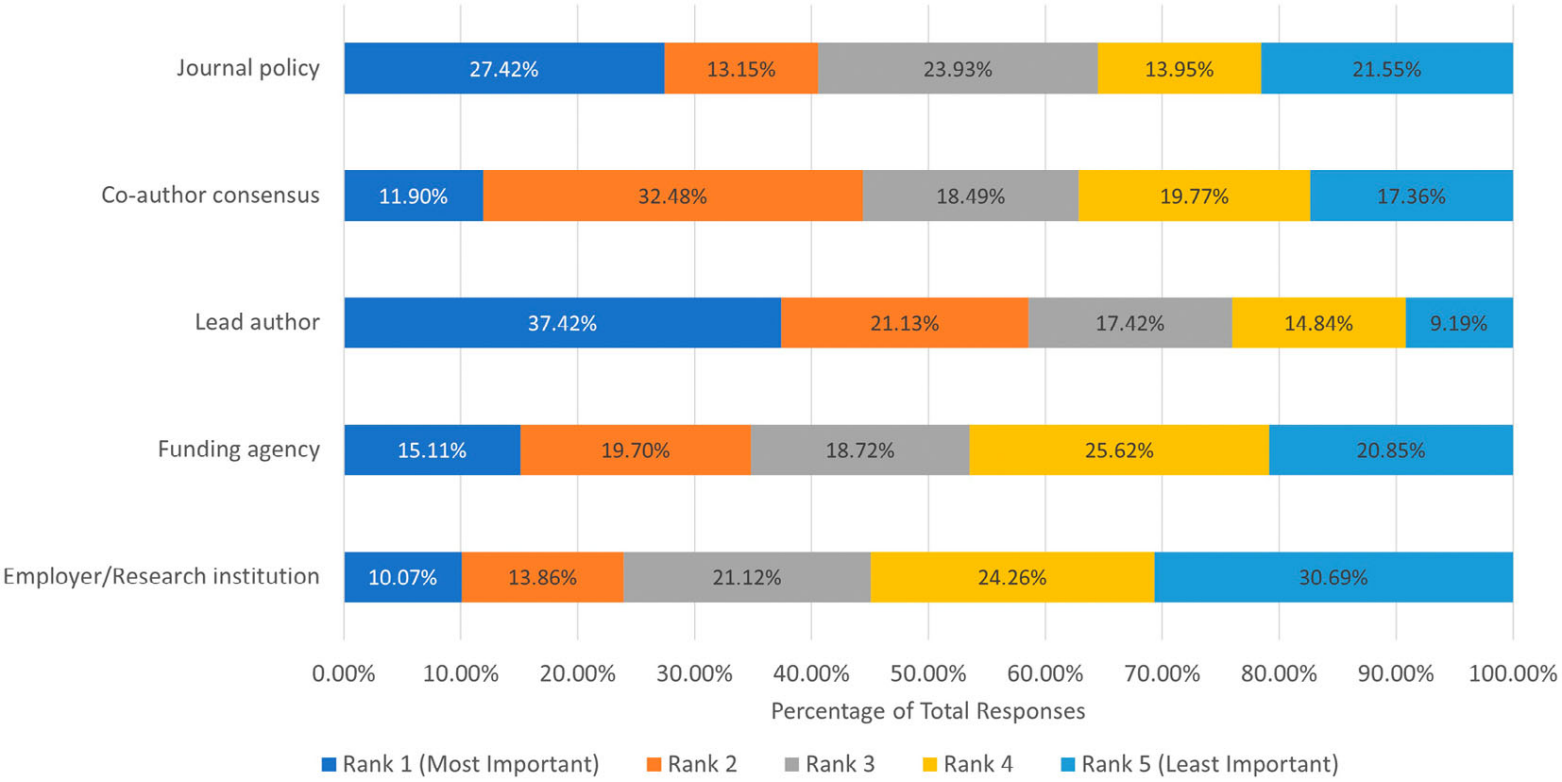
Ranking of factors that influence respondents’ decision to post a preprint.

We then aimed to determine the factors that impact an author’s decision to post a preprint. We asked participants to assess which had the most significant impact on posting a preprint: employer/research institution, funding agency, lead author, co-author consensus, and journal policy. From the 651 respondents, the lead author had the most significant impact on the decision of posting a preprint, followed by journal policy, co-author consensus, funding agency, and the least impactful is the employer/research institution ( Figure 1).

We also asked whether employers required or prohibited the posting of a preprint prior to submission to a journal. Most employers did not, with 649/690 (94.06%) respondents stating that their employer did not require them to post a preprint, and 657/691 (95.08%) respondents stating that employers do not prohibit them from doing so. In addition, we wanted to see if a funder may impact this decision, and 639/692 (92.34%) participants stated that funders do not require them to post a preprint.

**Figure 2.**
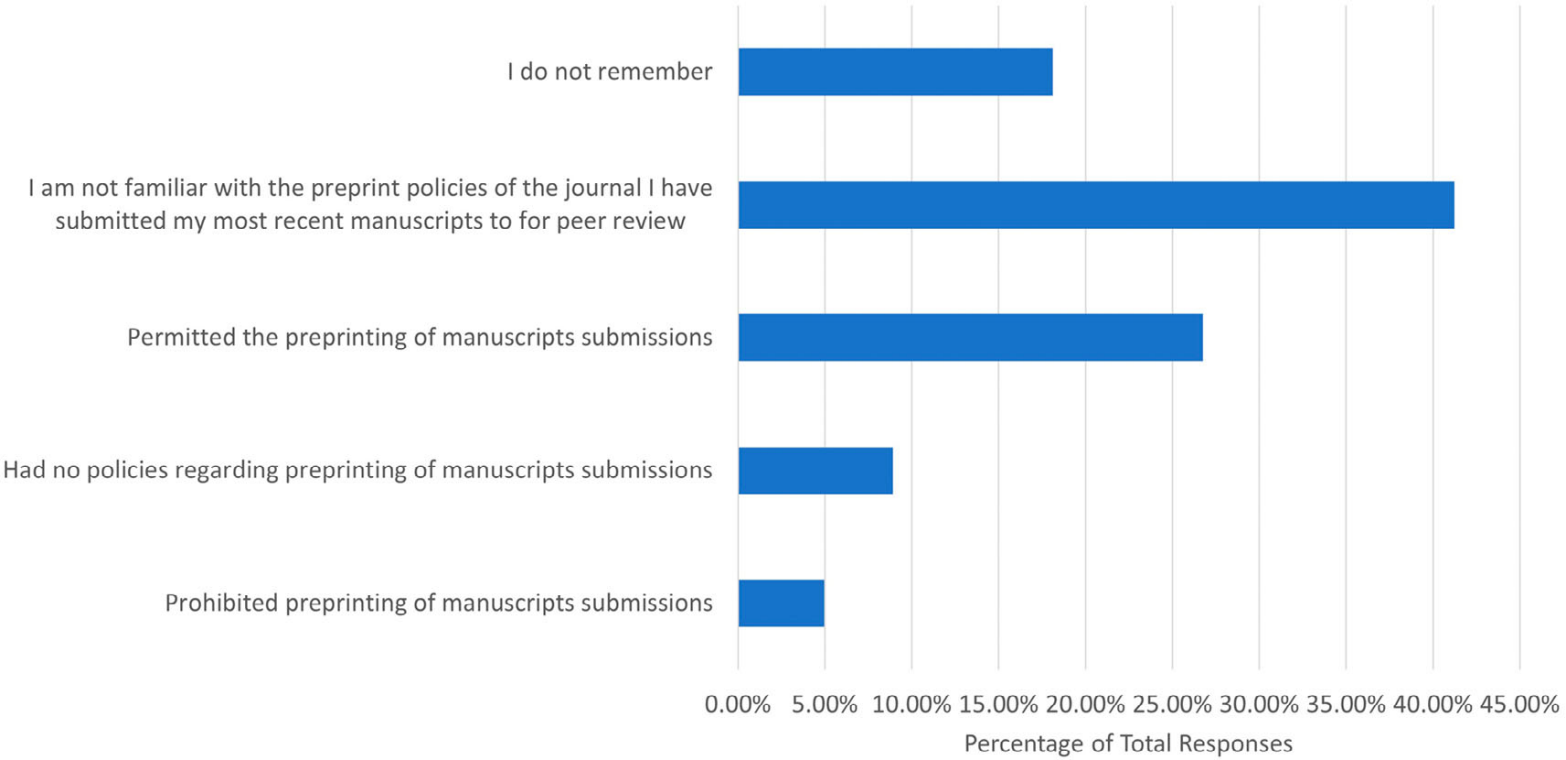
Preprint policies of journals respondents had most recently submitted a first author/corresponding author publication.

We also wanted to determine how familiar participants were with the preprint policies of journals that they had experience of publishing in. First, we asked whether participants were familiar with either Sherpa Romeo or Transpose Publishing, which are resources that present the preprint policies of most peer reviewed journals. Of the 686 responses, 623 (90.95%) were not familiar with Sherpa Romeo, and 656 (95.91%) were not familiar with Transpose Publishing. In addition, we asked what the preprint policy was for the journals of respondents’ most recent first author/co-author publications ( Figure 2). Of the 684 responses, 282 (41.23%) stated that they were not familiar with the preprint policies, while 183 (26.75%) stated that preprinting was permitted. Only 34 (4.97%) respondents and 61 (8.92%) respondents stated that preprinting was prohibited or that they had no preprinting policies, respectively.

**Figure 3.**
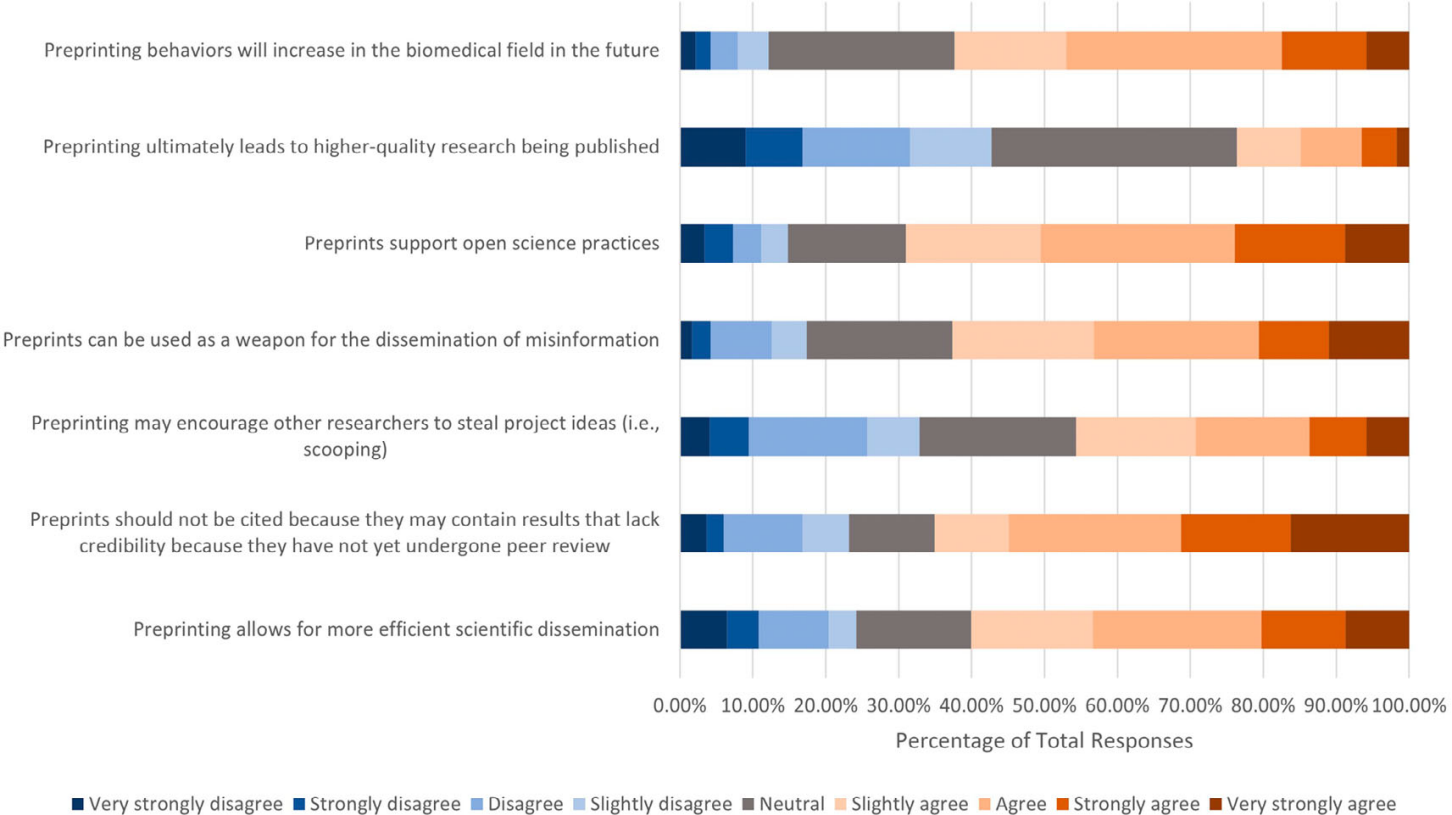
Respondents’ opinions on incentives and consequences of preprinting.

Respondents were asked to what extent they agreed with certain incentives or the consequences of preprinting ( Figure 3). Of the 666 who answered, over half had positive attitudes toward the following incentives and consequences: 1) Preprints support open science practices (n=456, 68.99%); 2) Preprints should not be cited because they may contain results that lack credibility because they had not yet undergone peer review (n=433, 65.12%); 3) Preprints can be used as a weapon for the dissemination of misinformation (n=417, 62.61%); 4) Preprinting behaviors will increase in the biomedical field in the future (n=415, 62.32%); and 5) Preprinting allows for more efficient scientific dissemination (n=400, 60.06%). In terms of whether respondents agreed or disagreed with the statement “Preprinting may encourage other researchers to steal project ideas (i.e., scooping), less than half of (n=304, 45.66%) respondents agreed, 143 (21.47%) respondents remained neutral, and 219 (32.89%) respondents disagreed. On the other hand, 284 respondents (42.71%) did not agree that preprinting would ultimately lead to higher-quality research being published, while 224 (33.68%) and 157 (23.60%) respondents chose neutral and agreement options, respectively.

**Figure 4.**
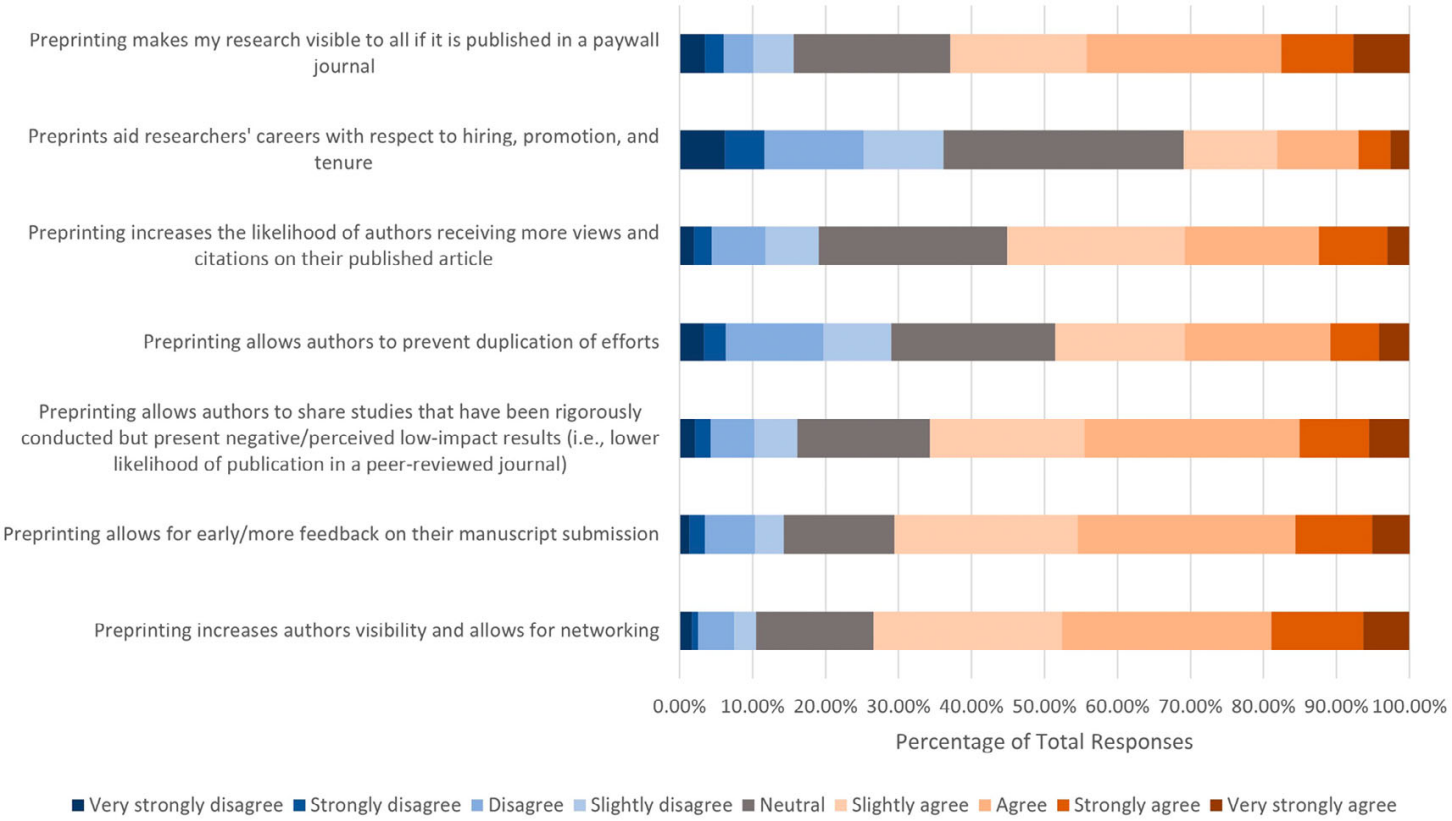
Respondents’ opinions on motivations to publish a preprint.

We then asked the extent to which respondents agreed with the following motivations for publishing a preprint ( Figure 4). Of the 666 respondents who answered, over half had positive attitudes toward the following incentives and consequences: 1) Preprinting increases authors’ visibility and allows for networking (n=489, 73.43%); 2) Preprinting allows for early/more feedback on their manuscript submission (n=470, 70.58%); 3) Preprinting allows authors to share studies that have been rigorously conducted but present negative/perceived low-impact results (i.e., lower likelihood of publication in a peer reviewed journal) (n=437, 65.70%); 4) Preprinting makes my research visible to all if it is published in a paywall journal (n=415, 62.89%); and 5) Preprinting increases the likelihood of authors receiving more views and citations on their published article (n=365, 55.14%). In addition, slightly less than half of respondents (n=323, 48.57%) agreed that preprinting allows authors to prevent duplication of efforts, while 193 (29.02%) disagreed, and 149 (22.42%) remained neutral. In terms of if respondents agreed or disagreed with the statement “Preprints aid researchers’ careers with respect to hiring, promotion, and tenure”, respondents’ opinions were more evenly split, with 205 (30.91%) respondents agreeing, 218 (32.88%) respondents remaining neutral, and 240 (36.19%) respondents disagreeing.

In addition, we asked participants for their opinions on making preprinting mandatory during the research process. Of the 665 respondents who expressed opinions, over half (n=363, 54.58%) were leaning toward disagreeing with the statement. Only approximately a quarter (n=163, 24.52%) of the respondents had positive opinions regarding this statement.

We then asked an open-ended question to allow respondents to freely express other factors that motivated or dissuaded them from preprinting ( Table 3). The 197 responses could be primarily split into positive and negative factors. The most prevalent positive factor that motivated preprinting “Preprints allow rapid dissemination”. This was followed by “Preprints show research efforts,” in which preprinting allowed authors to show their research to other parties, such as other researchers, funding agencies, and universities, without the use of a publication in a journal. Lastly, the final positive factor commonly mentioned was “Preprints are open-access items”.

**Table 3.** Thematic analysis of the open-ended question (n=197).

Themes	Subthemes	Representative quote	Number of responses
Factors that negatively influences factors to preprint			
	Peer review is bypassed	“The regular method of disseminated research provides quality control in the form of peer review. Preprint often does not have this, but looks the same as a publication. […]”	63
	Preprinting is not needed	“Preprint publications are not considered in scientists production evaluations at my Institution.”	26
	Preprints might release misinformation	“Not peer reviewed results in preprints are often treated like solid scientific evidence in the media. This could be very harmful.”	25
	Preprints are poor quality research	“In my experience, I have seen very terrible research on preprint servers. […] The potential for applying erroneous/poor research presented in preprint manuscripts is much higher than regular peer reviewed journals.”	24
	Journal policy impacts preprinting decisions	“Worries as regards a potential negative influence on the chance of acceptance/editorial judgement – in spite of a journal policy of no objections to preprint.”	20
	Preprinting requires additional resources	“Another layer of administrative work to create an account, upload. Not clear if there is any benefit. […] Seems like duplication of effort.”	17
	Scooping	“I am hesitant and based on my experience to preprint publish work, which can be easily replicated and published before our own. […]”	17
	Unfamiliar with preprints	“Never done it before and can’t think of a fellow researcher who has done it who would/could help me become familiar with it/normalize it.”	15
	Multiple versions of the same paper	“The preprint never gets removed form the site once the peer reviewed publication is available. This is confusing because several references refer to the same published data. The data may change after peer review (e.g. additional studies may report information that is pertinent to the interpretation of the data in the Preprint.”	13
Factors that positively influenced decisions to preprint			
	Preprints allow rapid dissemination	“The peer review process is too slow to rely on it or the efficient sharing of scientific results”	23
	Preprints show research efforts	“I have posted research this is close to a final/published version. This was to get our research out to the community in a more timely manner and to demonstrate to my field that we have done this research.”	16
	Preprints are open access items	“I have never published a preprint before. But having limited access to full-text articles motivates the use of preprint sources.”	10

On the other hand, the open-ended questions showed that many negative factors discouraged respondents from preprinting. The most frequent, with approximately a third of responses, was “Peer review is bypassed,” as many responses state that peer review must be carried out to show credibility and quality of research before publishing. In addition, many responses believed that “Preprinting is not needed,” since preprinting does not provide any benefits to the individual’s career and there were no issues with the current publishing system. Another negative factor was “Preprint might release misinformation,” followed by “Preprints are poor-quality research.” Next, many responses stated that the journal policy prevented preprinting”, since journal policy rejected or was unclear about the preprints. “Scooping” was also mentioned frequently, along with “Preprinting requires additional resources,” where respondents felt that preprinting needed extra time, effort, and money that is unnecessary for the overall publication of their work. Additionally, many respondents were “Unfamiliar with preprints” which discouraged them from preprinting. Lastly, “Multiple versions of the same paper” was another negative factor, where having a preprint and a journal published version of the same paper results in the release of unedited, possibly incorrect, work and causes confusion when referencing.

## Discussion

The objective of this study was to explore biomedical researchers’ attitudes toward preprints to determine what factors influence their opinions about publishing and viewing preprints. We found that the most impactful preventative factors against preprinting were the fear of disseminating misinformation, lack of peer review, and unsupportive journal policies. While hiring, promotion, and tenure do not encourage preprinting, respondents believed that preprints are beneficial in increasing visibility and recognition, being openly accessible, providing rapid feedback, and publishing negative data.

Future work may focus on determining how to improve researchers’ attitudes toward preprinting and how other stakeholders can implement and modify their preprint policies in the future. To the best of our knowledge, this cross-sectional survey is the first to focus exclusively on biomedical researchers to explore their attitudes toward preprinting. The results of this approach are essential for gaining a clearer understanding of the perspectives that biomedical researchers have on preprints, which can potentially impact how other researchers, institutions, and publishing houses implement preprint policies in the future to improve preprinting in biomedical researchers.

### Opinions on the benefits and consequences of preprints

In terms of the benefits and consequences of preprints, the three statements that respondents most agreed with were: 1) Preprints support open science practices, 2) Preprints should not be cited because they may contain results that lack credibility because they have not yet undergone peer review, and 3) Preprints can be used as a weapon for the dissemination of misinformation. These results align with the preliminary work done by Funk *et al*. ( https://asapbio.org/), who found that the most concerning issues with preprints from the perspective of respondents were premature media coverage and public sharing of information prior to peer review, while respondents believed that the top benefit of preprints is open access.

On the other hand, the top three most impactful factors among respondents with respect to deciding to publish a preprint included 1) increasing author visibility and networking opportunities, 2) allowing for early and more feedback on manuscripts, and 3) publishing of negative or perceived low-impact results. The findings by Funk *et al*. somewhat align with our results, as they found that only over half of the respondents found early feedback as “very beneficial” and were only seen as less impactful in comparison to open access and speed of dissemination. However, publishing negative or perceived low-impact results was the second least impactful factor, only being seen as “very beneficial” by approximately one-third of the respondents. This variation may be due to more awareness of the bias of journals toward positive results, and why this is an issue for the scientific community. Oftentimes, authors aim to only publish positive “groundbreaking” results, and journals often reject papers with negative or non-significant results to increase readers, citations, and submissions. ^14^^,^^15^ However, negative and non-significant results are vital to the scientific process as they allow for collective self-correcting progress and ensure that resources are not wasted on the replication of failed research. ^15^^,^^16^ A survey by Echevarría *et al*. ^17^ showed that the majority of authors believe this information should be shared, although only a handful have tried to publish negative results. Preprints may be an appropriate alternative for publishing negative data as they can quickly share this information without a long peer review process, there is no chance of the manuscript being rejected, and there are no concerns with journal metrics. Future work should focus on gaining a better understanding of researchers’ attitudes toward publishing negative results and preprint practices to ensure that negative results are appropriately disseminated within the scientific community.

One hesitation toward the use of preprints revolves around the fact that preprints have not undergone peer review; therefore, the scientific content has not been validated before being released to the public. This is seen in our findings, in which almost two-thirds of our respondents believe that preprints should not be cited because the results may lack credibility since there was no peer review. Moreover, over two-thirds of our respondents had viewed or downloaded a preprint, but only a quarter had ever cited it, implying distrust in preprint content. Additionally, peer review being bypassed was the most frequent factor that negatively impacted respondents’ motivations to preprint, as seen in the open-ended question, alongside the fear that preprints will disseminate misinformation and poor-quality research. Thus, we consistently observed that peer review is regarded as highly important and necessary for publishing research by respondents. Respondents’ hesitation toward preprints due to lack of peer review and fear of misinformation indicated a lack of knowledge about the evidence regarding the quality of the published literature and the effectiveness of peer review. Work conducted by Zeraatkar *et al*. ^18^ has shown that there was no evidence that preprints provided results that were inconsistent with peer reviewed publications, specifically seen with COVID-19 treatments. MEDLINE is also currently indexing preprints, supporting research that has been posted to eligible preprint servers for easy discoverability and preservation ( https://www.ncbi.nlm.nih.gov/). In addition, it is important to note that peer review is not always effective because of inconsistency and bias, as many peer reviewers have not had standard training, and many training opportunities and courses are not openly accessible online, inhibiting researchers from completing training on peer review. ^18^^–^^22^ Thus, this blind belief in peer review may negatively impact researchers’ opinions toward preprints.

Interestingly, despite many respondents distrusting preprints due to a lack of peer review, many were divided on whether peer review should become part of the preprint process. This may be because preprints allow for the rapid dissemination of research as there is a delay from journal submission to publication when undergoing peer review. ^1^^,^^5^ If peer reviews were to be incorporated into preprinting in the same way as it was done for journals, then the work of peer reviewers would increase and the delay would become much longer. Thus, one suggestion may be to modify preprint servers in such a way that they improve the credibility of preprints, as suggested by Soderberg *et al*. ^5^ The current preprint feedback system allows for public feedback and discussion, which may aid in credibility or results. However, authors often fear unfair criticism from competitors, harm to their reputation, or softened criticism due to the public nature of feedback. ^7^ Fortunately, the FAST principles can alleviate these issues. ^6^ From our findings, the majority of respondents had not peer reviewed a preprint before, and of those that had, most were not familiar with the FAST principles. Therefore, efforts must be made to educate researchers on these principles to promote feedback on preprints. Future work may aim to better understand researchers’ opinions on peer reviewing preprints and potentially provide other novel solutions such that the benefits of peer review can be implemented in the preprinting process without the current fears of preprint feedback.

### External factors that play a role on opinions and attitudes toward preprints

Journal policy is the second most impactful factor in the decision of authors to post a preprint and was frequently mentioned in the open-ended question. In addition, most respondents were unfamiliar with the journal’s preprint policy. Nowadays, most, but not all, life science journals accept preprinted manuscripts for submission, and some journals either do not have a preprint policy or have contradicting statements. ^3^ Biomedical journals should create clear and concise preprint policies that allow researchers to understand whether they can publish preprints before or during submission to a journal. In addition, our data showed that most researchers do not know of resources on preprint policies, such as Sherpa Romeo and Transpose Publishing, which gather journal policies on open access and preprinting. In the future, it would be best to educate researchers on these types of resources to address the concerns that preprints negatively impact the chances of publishing in a peer reviewed journal.

The respondents did not have strong opinions on whether preprints aid researchers’ careers with respect to hiring, promotion, and tenure. In addition, our findings showed that universities and research institutes had the least impact on one’s decision to publish a preprint, and the majority of our respondents’ employers neither prohibited nor required the posting of preprints. This may be because few academic institutions/universities consider preprints when hiring and for promotion ( https://asapbio.org/). Therefore, hiring, promotion, and tenure policies should be modified, as current methods rely heavily on quantitative metrics, which have been recognized as a flawed system. ^23^ Preprints may prevent the hiring committee’s bias toward or against a journal due to a journal’s impact factor or reputation ^24^ ( https://sfdora.org/). The inclusion of preprints in hiring, promotion, and tenure policies may improve biomedical researchers’ attitudes toward preprinting.

### Strengths and limitations

By implementing a cross-sectional survey for this project, we took a snapshot of the population of interest without having to follow them over time. This study’s findings are arguably highly generalizable across biomedical researchers because we randomly surveyed a large sample of biomedical researchers with varying opinions regarding preprint postings. A limitation of our study design was that it was written in English, so researchers without working knowledge of English could not take the survey. In addition, as the present study was based on an English-speaking international sample, it does not consider the impact of national policies on attitudes and opinions toward preprinting. Another limitation of our sampling strategy is that the list of email addresses used potentially included inactive or invalid addresses, which may have been a result of changing professions, retiring, or passing away. Furthermore, we did not consider autoreplies. Therefore, the response rate is underestimated. Our questionnaire was built on self-declared attitudes and preprint practices, which may not accurately capture independently confirmed practices. Inherent to the cross-sectional survey design, the final limitations also include a low response rate, recall bias, in which participants do not correctly remember past events, and non-response bias, in which participants did not want to or could not complete a survey question. In addition, responses mostly reflected the opinions of senior academics, we there were far less participants who were early career researchers, as a result of our sampling strategy.

## Conclusions

In this study, we surveyed biomedical researchers about their knowledge, experiences, and attitudes toward preprinting. We found that biomedical researchers were familiar with the concept of preprints but lacked experience in working with preprints. The various attitudes and opinions of biomedical researchers provide valuable contributions to the field of publication science and suggests that a number of changes are warranted in the scientific community. To our knowledge, this is the first study to examine the attitudes and opinions of preprints in a discipline-specific manner. Therefore, this study can be used as a model for other academic researchers from alternative disciplines who may be interested in preprinting. Additionally, it would be of value to determine attitudes and opinions in other disciplines to find methods to improve preprinting and researchers’ attitudes toward it. By identifying the attitudes of biomedical researchers towards preprinting, this study’s findings can inform the provision of suggestions to relevant stakeholders for the implemention and improvement of preprinting.

## Consent

Written informed consent for publication of the participants’ details was obtained from the participants.

## Data Availability

Open Science Framework: Underlying data for ‘An international, cross-sectional survey of preprint attitudes among biomedical researchers’, https://doi.org/10.17605/OSF.IO/QA9GN. ^12^ This project contains the following underlying data:
• Preprints Survey Raw Data_Sep1723.xlsx • Current Career Stage_Sep1723.docx • Employment Sector_Sep1723.docx • Gender_Sep1723.docx • Participants’ Research Area_Sep1723.docx Open Science Framework: Extended data for ‘An international, cross-sectional survey of preprint attitudes among biomedical researchers’, https://doi.org/10.17605/OSF.IO/QA9GN. ^12^ This project contains the following extended data:
• Preprints Survey Protocol_Oct0322.pdf • Preprints Survey_Coding and Themes_Sep1723JYN.xlsx • Supplementary File 1: Search strategy • Supplementary File 2: Survey Open Science Framework: CHERRIES checklist for ‘An international, cross-sectional survey of preprint attitudes among biomedical researchers’, https://doi.org/10.17605/OSF.IO/QA9GN. ^12^ Data are available under the terms of the Creative Commons Attribution 4.0 International license (CC-BY 4.0).
