# Supplementary material for: An international, cross-sectional survey of preprint attitudes among biomedical researchers
Source: F1000Res. 2024 Nov 4;13:6. Originally published 2024 Jan 3. [Version 2] doi: 10.12688/f1000research.143013.2 (PMC11589411; doi:10.12688/f1000research.143013.2)
Supplement: Peer review history

# Reviewer report: Reviewer response for version 2

**DOI:** 10.5256/f1000research.173500.r342534

**Published:** 2025-01-03

**Authors:** Andrew Tattersall

**Affiliations:** 1 University of Sheffield, Sheffield, England, UK

The one notable issue for me relating to this paper is the clarity relating to the title and the cohort. As it cannot be gleaned as to whether all of these researchers are biomedical researchers, the title and subsequent text perhaps should be changed. From 'biomedical researchers' to 'researchers publishing in biomedical journals'. As it is likely some will be based in social sciences etc. and would not consider themselves biomedical academics. It is a shame that this was not picked up via the questionnaire to weed out non biomedical researchers. I appreciate that if publishing a paper that is indexed in MEDLINE may muddy the water somewhat, but it would have been good to have captured what those in the sample referred to themselves as. 

 I have nothing else to add other than the other reviewers have highlighted in their open comments. I think it is an interesting piece of work and the discussion highlights the flaws of this study and where subsequent work could be undertaken to carry out further research into the opinions and experiences of researchers and preprints.

Is the work clearly and accurately presented and does it cite the current literature?

Yes

If applicable, is the statistical analysis and its interpretation appropriate?

Yes

Are all the source data underlying the results available to ensure full reproducibility?

Yes

Is the study design appropriate and is the work technically sound?

Yes

Are the conclusions drawn adequately supported by the results?

Yes

Are sufficient details of methods and analysis provided to allow replication by others?

Yes

Reviewer Expertise:

Information Science, Open Research, Research Communication

I confirm that I have read this submission and believe that I have an appropriate level of expertise to confirm that it is of an acceptable scientific standard, however I have significant reservations, as outlined above.

# Reviewer report: Reviewer response for version 2

**DOI:** 10.5256/f1000research.173500.r342532

**Published:** 2024-12-11

**Authors:** Shelly Melissa Pranic

**Affiliations:** 1 Cochrane Croatia, Split, Croatia

Elucidating factors surrounding the use of preprinting to disseminate scientific findings is important. Biomedical research is constantly growing, and researchers' perceptions about preprinting provide information about future usage of preprinting. Although the number of survey respondents varied across questions, this cross-sectional study could provide information for future studies. Do the authors have plans to follow up with the authors who are unsure about submitting or intend to submit a preprint in the future? It would be interesting to see if their intention is associated with actual preprinting. 

 Please find my comments according to the article section regarding more information about some methodological aspects of the study. Thank you.  

 Methods

 Sampling framework

 1. Why was the period 2021/07/01 to 2022/08/01 chosen to search for published articles? Data management and analysis

 2. Regarding the thematic content analysis, how many research members carried out the coding? Can you list these members?

 3. For which coded category was there the least agreement between the team members?

 Discussion

 Strengths and limitations

 1. Regarding the non-response to some of the questions, which question had the least number of responses? Maybe this could be mentioned in the results section to inform readers.

Is the work clearly and accurately presented and does it cite the current literature?

Yes

If applicable, is the statistical analysis and its interpretation appropriate?

Not applicable

Are all the source data underlying the results available to ensure full reproducibility?

Yes

Is the study design appropriate and is the work technically sound?

Yes

Are the conclusions drawn adequately supported by the results?

Yes

Are sufficient details of methods and analysis provided to allow replication by others?

Yes

Reviewer Expertise:

Health services research, epidemiology, and public health.

I confirm that I have read this submission and believe that I have an appropriate level of expertise to confirm that it is of an acceptable scientific standard, however I have significant reservations, as outlined above.

# Reviewer report: Reviewer response for version 2

**DOI:** 10.5256/f1000research.173500.r342537

**Published:** 2024-12-11

**Authors:** Amanda Blatch-Jones

**Affiliations:** 1 University of Southampton, Southampton, UK

Thank you for the opportunity to review the revised manuscript (version 2). The authors touch upon a relevant and important area in the field of research and understanding the views of researchers is important to the use and acceptability of preprints.

 Having read version 1 and version 2, I have a few comments that I have listed below related to version 2:

 Introduction

- The authors state that peer review is not always effective. Explaining this in more detail would be advantageous for the reader.
- Elaborating on how institutions and funders are becoming more open about their policies on preprints would strengthen this statement, along with the evidence to support this. Although some funders do, it is very much funder and country specific, and in most cases relates to the final reporting of research and not for grant applications or applicants CVs (a review on preprints from funding organisations was carried out and there was minimal information about the use of preprints in grant applications and applicants CVs - Blatch-Jones et al 2023 Ref 1)
- There is a difference across disciplines as to the potential harm and use of preprints, although this may be particularly relevant in health and social care disciplines, it is less so in other disciplines such as engineering, and non-health related research fields. It would be important to mention the diversity as this does have impact on the acceptability and use of preprints (as well as the views from researchers)
- As this manuscript is specifically related to biomedical researchers, the concerns and hesitancy on the use of preprints could to be drawn out more in the introduction, At the moment it is written more to the positive angle of preprints rather than the challenges, drawbacks and concerns to their use

Methods

- More information is required on how the thematic content analysis was carried out, what approach did you use (framework, deductive, inductive), was the thematic content analysis piloted and how was the results analysed (in Excel or other software) and themed?
- There is minimal information related to the thematic content analysis that you would expect from using this approach. It might be worth visiting some examples to determine if this was the approach you used or not.
- The methods section provides sufficient details about the sample, although clarification on the date range 2021 - 2022 would explain why it is more than two years ago. In the last two years preprints have been a focal point of discussion and views and opinions have changed across disciplines (people are more willing to explore the use of preprints whilst also drawing attention to the caveats and concerns when using or reporting their use)

Results

- I do not have any comments about the results section. It is well written and the headings are sufficient to explain the results of the study.

Discussion

- The authors state 'examine how other stakeholders can adjust their preprint policies in the future' - who do you mean and to what extent? Some funding organisations do not have a preprint policy or guidance and academic institutions may have different position statements on their use. This is also the case for publishers, some promote and encourage the use whereas others do not have any position statement. I feel the authors need to draw this out more and align more to the literature in this space as well.
- Referring back to the literature to show how this study addresses previous conclusions (e.g., Blatch-Jones et al 2023) but also how the findings complement what is already known (referring back to the literature in the introduction)
- Some of the references don’t appear to match the statements in the discussion and there are new references (and topic areas) that are not mentioned in the introduction (tenure, early career researchers etc). I would suggest picking these up to ensure a balanced account is threaded throughout the manuscript
- The section on benefits and consequences of preprints is difficult to read. I would suggest the authors take each point in turn as it would make these more readable and make it a balanced viewpoint (suggest discussing benefits and then consequences). However, is the term consequences the right word here? Would it be more balanced if it was challenges or barriers?
- The external factors have an important role to play for preprints, particularly around how and when it is acceptable to use preprints and the caveats around their use in medical disciplines. I feel that the authors could benefit from picking this up more and providing an equal balance with benefits and challenges?
- As the cohort were only biomedical researchers, this is a limitation. Although the authors mention this, it needs to be presented more explicitly as this is not the view for other disciplines, including those that have used preprints for several years, and those that rely on preprints to promote their research due to publication charges and open access.

Overall, the manuscript adds to the current evidence-base on preprints and provides a structured viewpoint from biomedical researchers that can be addressed/considered in future policy and guidance from funding organisations and publishers.

Is the work clearly and accurately presented and does it cite the current literature?

Partly

If applicable, is the statistical analysis and its interpretation appropriate?

Not applicable

Are all the source data underlying the results available to ensure full reproducibility?

Yes

Is the study design appropriate and is the work technically sound?

Yes

Are the conclusions drawn adequately supported by the results?

Partly

Are sufficient details of methods and analysis provided to allow replication by others?

Partly

Reviewer Expertise:

Research on Research, metascience, scoping reviews, peer review and decision-making, research management processes and practices, open research, qualitative research (observations, netnography). post-award management

I confirm that I have read this submission and believe that I have an appropriate level of expertise to confirm that it is of an acceptable scientific standard, however I have significant reservations, as outlined above.

## References

[1] : The use and acceptability of preprints in health and social care settings: A scoping review. *PLoS One* .2023;18(9) : 10.1371/journal.pone.0291627 e0291627 10.1371/journal.pone.0291627 37713422 PMC10503772

[2] : To preprint or not to preprint: A global researcher survey. *Journal of the Association for Information Science and Technology* .2024;75(6) : 10.1002/asi.24880 749-766 10.1002/asi.24880

# Reviewer report: Reviewer response for version 2

**DOI:** 10.5256/f1000research.173500.r337456

**Published:** 2024-11-25

**Authors:** Artemis Chaleplioglou

**Affiliations:** 1 Archival, Library & Information Studies (ALIS), University of West Attica, Aigaleo, Greece

The amendments applied clarify the methodology and issues.

Is the work clearly and accurately presented and does it cite the current literature?

Yes

If applicable, is the statistical analysis and its interpretation appropriate?

Yes

Are all the source data underlying the results available to ensure full reproducibility?

Partly

Is the study design appropriate and is the work technically sound?

Partly

Are the conclusions drawn adequately supported by the results?

Yes

Are sufficient details of methods and analysis provided to allow replication by others?

Partly

Reviewer Expertise:

Library and information science (LIS), Bibliometrics, Health information

I confirm that I have read this submission and believe that I have an appropriate level of expertise to confirm that it is of an acceptable scientific standard.

# Reviewer report: Reviewer response for version 2

**DOI:** 10.5256/f1000research.173500.r339638

**Published:** 2024-11-25

**Authors:** Luka Ursić

**Affiliations:** 1 University of Split School of Medicine, Split, Croatia

Many thanks for this opportunity to review the article by Ng and colleagues. I believe it is very topical and that it could help further our understanding of the uptake and perceived issues with preprints. I offer some remarks and suggestions below, grouped by specific article sections.

**General remarks**

 I believe a more tentative approach might be required in the article when commenting on preprints in the Abstract, Introduction, and Discussion sections. The authors take an overtly positive, optimistic approach to preprints as a publishing format; for example, by saying that ‘Uptake is not optimal’ in the Abstract, or highlighting that ‘Future work is needed to determine optimal ways to improve researchers’ attitudes through modifications to current preprint systems and policies.’ I think this automatically assumes that preprints are something that needs to be adopted, irrespective of any shortcomings, as if there is a general consensus within the scholarly community on this topic. I suggest that this is rephrased thoughtfully throughout the manuscript, or that the authors highlight this as their own stance, rather than one that is simply understood as normal.

**Introduction**

 There’s a few statements that I think should be contextualized. The first is the following: ‘While preprints may face scrutiny for not undergoing peer review, it is important to note that peer review is not always effective. ^6^’

 ‘May face scrutiny’ might be a bit of an understatement, as this is the main shortcoming of preprints overall. We do know that peer review is not ideal and has many shortcomings, but we do not have (as of yet) any viable alternatives. In any case, how do preprints servers help with this ineffectiveness? How does peer review then compare to comments left on these preprints? See these papers, which might be relevant:

 Malicki M, et.al., Ref 1 

 Carneiro CFD, et.al. Ref 2.

 The following statement is also a bit troublesome: ‘Preprints have become valuable for authors, as a larger group of researchers can critique their work (i.e., open peer review) via the preprint servers’ feedback system that allows for public and open feedback directly onto a manuscript that encourages discussion. ^7^’

 It is yet unclear how much commenting is being done for preprints. Preliminary studies suggest that this is not a really common practice, and that these comments generally are not fully-fleshed out peer reviews. See this study and the research cited within it: Malički M,  2021 Ref 3.  Here the situation seems a bit better, and the comments more robust, but still rare: Carneiro CFD, et.al., 2023 Ref 4

 The authors here state that preprints have become ‘valuable for authors’, but the reference cited in the manuscript does not necessarily support this. This scoping review might be useful to contextualise this: Blatch-Jones AJ Ref 5. Also citing the already cited study by Soderberg and colleagues would be more appropriate Ref 6

 In addition, some of these studies could help them contextualise this introduction section and highlight additional challenges that emerge with preprints, such as this one by Adarkwah and colleagues Ref 7 on AI and preprints or this one on the inclusion in systematic reviews: Brietzke E, et.al., 2023 Ref 8 

**Methods/Results**

 The methodology of the study is clear, so I have minimal suggestions, First, perhaps the authors should elaborate on why they selected 400 journals specifically; was there some sample size calculation here, or was this only a random decision? What was your expected response rate? Why were articles published between 2021/07/01 and 2022/08/01 only considered (i.e. why were only their authors contacted)?

 Second, I’d advise against using the phrase ‘thematic content analysis’ as it refers to a set of diverse approaches to qualitative analysis which are generally more structured and robust. For example, you do not detail your coding approach (i.e. was it based on a specific frameworks), if the ‘codes’ pre-determined or if they emerged from the analysis, etc. From your results, we can see that you again relied on the frequency of specific codes to draw inferences, which would go against some of the proposed thematic content analysis approaches. I think using the phrase ‘simple’ or ‘unstructured’ content analysis might be better, as this analysis was not as key to your study as your quantitative one and was rather supplemental.

 The results section is otherwise fine, and presented clearly and in detail. No suggestions there.

**Discussion**

 Here again I don't agree with the authors’ approach towards peer review. Two-thirds of their respondents have highlighted that they are concerned about a lack of peer review for preprints, which you only interpreted as them being unaware of the evidence in favour of preprints being as good as peer-reviewed articles. I am aware that existing studies have suggested this to be true, even the ones that found peer-reviewed articles to have higher quality of reporting, such as the one by Carneiro and colleagues which I linked above. However, this only speaks to preprints that have been peer reviewed and published. It does not necessarily address their concern, which might be related to the large number of preprints that are never reviewed or published or commented on (see above studies by Carneiro et al. and Costello et al.), but are still accessible to researchers and the public, and therefore may contain unverified information which could be misinterpreted or misused. For example, even medRxiv ( https://www.medrxiv.org/) suggests that preprints should not be used to inform clinical practice or health-related behaviors explicitly because they are not peer reviewed, and that they should not be reported in the news. You do highlight this in your introduction, but could expand on it here as a sort of caveat for the discussion, speaking of issues on media picking up preprints, or AI using them for viable sources, or their uptake in systematic reviews. Therefore, I think this should be expanded to feature both the positives and the negatives of the preprint/peer review relationship.

 I hope these comments will help the articles in improving their articles and adding the necessary nuances on this subject.

Is the work clearly and accurately presented and does it cite the current literature?

Partly

If applicable, is the statistical analysis and its interpretation appropriate?

Yes

Are all the source data underlying the results available to ensure full reproducibility?

Yes

Is the study design appropriate and is the work technically sound?

Yes

Are the conclusions drawn adequately supported by the results?

Partly

Are sufficient details of methods and analysis provided to allow replication by others?

Partly

Reviewer Expertise:

Meta-research.

I confirm that I have read this submission and believe that I have an appropriate level of expertise to confirm that it is of an acceptable scientific standard, however I have significant reservations, as outlined above.

## References

[1] : Preprint Servers' Policies, Submission Requirements, and Transparency in Reporting and Research Integrity Recommendations. *JAMA* .2020;324(18) : 10.1001/jama.2020.17195 1901-1903 10.1001/jama.2020.17195 33170231 PMC7656281

[2] : Comparing quality of reporting between preprints and peer-reviewed articles in the biomedical literature. *Res Integr Peer Rev* .2020;5(1) : 10.1186/s41073-020-00101-3 16 10.1186/s41073-020-00101-3 33292815 PMC7706207

[3] : Analysis of single comments left for bioRxiv preprints till September 2019. *Biochem Med (Zagreb)* .2021;31(2) : 10.11613/BM.2021.020201 020201 10.11613/BM.2021.020201 33927548 PMC8047782

[4] : Characterization of Comments About bioRxiv and medRxiv Preprints. *JAMA Netw Open* .2023;6(8) : 10.1001/jamanetworkopen.2023.31410 e2331410 10.1001/jamanetworkopen.2023.31410 37647065 PMC10469270

[5] : The use and acceptability of preprints in health and social care settings: A scoping review. *PLoS One* .2023;18(9) : 10.1371/journal.pone.0291627 e0291627 10.1371/journal.pone.0291627 37713422 PMC10503772

[6] : Credibility of preprints: an interdisciplinary survey of researchers. *R Soc Open Sci* .2020;7(10) : 10.1098/rsos.201520 201520 10.1098/rsos.201520 33204484 PMC7657885

[7] : Are Preprints a Threat to the Credibility and Quality of Artificial Intelligence Literature in the ChatGPT Era? A Scoping Review and Qualitative Study. *International Journal of Human–Computer Interaction* .2024; 10.1080/10447318.2024.2364140 1-14 10.1080/10447318.2024.2364140

[8] : Should systematic reviews and meta-analyses include data from preprints?. *Trends in Psychiatry and Psychotherapy* .2023; 10.47626/2237-6089-2021-0324 10.47626/2237-6089-2021-0324 PMC1016440134551239

# Reviewer report: Reviewer response for version 1

**DOI:** 10.5256/f1000research.156628.r291988

**Published:** 2024-07-13

**Authors:** Kimberly Powell

**Affiliations:** 1 Emory University Woodruff Health Sciences Center Library, Atlanta, GA, USA

This study offers a broad survey of biomedical authors, identified by 12month sampling of MEDLINE publications, to summarize the awareness, attitudes, and use of preprints. The abstract claims findings can be used to assist stakeholders to more effectively implement preprinting behaviors, and an awareness of author landscape of attitudes and motivations is certainly required to inform more effective policies for support. However, the stated goal of the survey is clearly to increase preprinting and create a more favorable view of preprints rather than taking a more neutral perspective.

 While I do believe the survey to be well designed and executed, I do have some reservations about the presented framing of the study, as well as lack of formal citations supporting some areas of discussion. A significant portion of the discussion is dedicated to de-valuing peer-review which is not based in study data but rather the authors response to respondents perceived lack of awareness of concerns around the modern peer-review process. This particular framing would be better supported by introducing it in the introductory paragraphs and presenting preprints as a reasonable alternative so readers could be better prepared for this line of reasoning.

 Specific comments and suggestions are enumerated below:

1. Methods section open science statement offers a reference link for the OSF datafiles. Please also provide a reference link for the MedRxiv preprint.
2. The first paragraph of the introduction includes two parenthetical references to https://asapbio.org. With another instance in the third paragraph of the discussion. Additional context clues this reference in the discussion indicate that the proper citation should be made the ASAPbio blog page from 2020-07-27 available here: https://asapbio.org/biopreprints2020-survey-initial-results. All ASAPbio citations should be updated to formally appear in the reference list with their direct blog links. The references in the introduction do not offer enough information to track down the intended citations but should be updated to the appropriate reference information as well. A fourth orphaned ASAPbio reference is also made in the second paragraph of the “External Factors…” section of the discussion.
3. Final sentence of the Methods paragraph on Participant Recruitment predicts a 10% response rate. Since this is remarkably different the that 3.2% response rate, I wonder if the authors could provide any justification or rationale for the 10% prediction. Was this estimate based on similar author-response studies? Length/survey presentation/etc. Citations supporting this estimate would be appreciated for further research and study design.
4. I was confused by the inclusion of ResearchGate as a common preprint server to view/download preprints as presented in the Discussion section on Viewing/downloading preprints. Did 20.53% of respondents think downloading articles from ResearchGate equated to engaging with preprints? What about those cases where ResearchGate allows for downloading of the final publisher version? Can additional context around this survey question be provided to indicate how respondents thought ResearchGate related to preprints? I think to present it primarily as preprint server is a very concerning misunderstanding of the ResearchGate platform.
5. I was startled by the data presented in Table 2 and I do not think the introduction appropriately framed the issue of mandating peer review in the preprinting process as something that would even be asked in such a survey. When the first sentence of the manuscript offers a definition of preprint as something “which has not yet undergone formal peer review” the survey question of mandating peer-review become incongruous with the definition of what is being studied. Similarly, if authors wish to present trust in peer-review as a key takeaway of the survey results, concerns around peer-review, and the role of preprints as one possible alternative, would be better introduced in the introduction of the manuscript.
6. Table 3 respondent column should be presented as a percentage of the total responses for that question, as provided in all other figures.
7. The first paragraph of the discussion presents three main findings of the study that fear of misinformation, lack of peer review, and unsupportive journal policies were the most impactful preventative factors against preprinting. I am not convinced this is an appropriate representation of the data. While the first two points may indeed be primary concerns, I think unsupportive journal policies is an overstatement of the presented data which instead highlights respondents lack of awareness around journal policies and policy tools. While Figure 1 does show Journal Policy to be a more significant influence on choose to preprint than other factors, Figure 2 highlights that over 50% of authors aren’t familiar with journal policies at all or don’t remember the journal policies towards preprints for their last submissions. These data seem to support a lack of awareness on the part of the author, and not unsupportive journals. In fact, only 5% of authors are shown to be aware that their last journal for submission prohibited preprinting. The lack of author awareness is further discussed in the seventh paragraph of the discussion but the summarizing of this as “unsupportive journal policies” needs further justification. Relatedly, please add reference(s) to the claims that “most, but not all, life science journals accept preprinted manuscripts for submission” as this should be a key point of context.  

Is the work clearly and accurately presented and does it cite the current literature?

Partly

If applicable, is the statistical analysis and its interpretation appropriate?

Partly

Are all the source data underlying the results available to ensure full reproducibility?

Yes

Is the study design appropriate and is the work technically sound?

Yes

Are the conclusions drawn adequately supported by the results?

Partly

Are sufficient details of methods and analysis provided to allow replication by others?

Yes

Reviewer Expertise:

Librarianship; Scholarly Communications; Bibliometrics; Research Impact; Open Access Policies; Predatory Publishing

I confirm that I have read this submission and believe that I have an appropriate level of expertise to confirm that it is of an acceptable scientific standard, however I have significant reservations, as outlined above.

# Response

**Published:** 2024-10-15

**Authors:** Jeremy Ng

**Affiliations:** University of Ottawa, Canada

This study offers a broad survey of biomedical authors, identified by 12month sampling of MEDLINE publications, to summarize the awareness, attitudes, and use of preprints. The abstract claims findings can be used to assist stakeholders to more effectively implement preprinting behaviors, and an awareness of author landscape of attitudes and motivations is certainly required to inform more effective policies for support. However, the stated goal of the survey is clearly to increase preprinting and create a more favorable view of preprints rather than taking a more neutral perspective.

- We kindly thank the reviewer for providing their feedback on our manuscript.

While I do believe the survey to be well designed and executed, I do have some reservations about the presented framing of the study, as well as lack of formal citations supporting some areas of discussion. A significant portion of the discussion is dedicated to de-valuing peer-review which is not based in study data but rather the authors response to respondents perceived lack of awareness of concerns around the modern peer-review process. This particular framing would be better supported by introducing it in the introductory paragraphs and presenting preprints as a reasonable alternative so readers could be better prepared for this line of reasoning.

- We have made changes to the Background of the manuscript to reflect these suggestions.

Specific comments and suggestions are enumerated below:

1. Methods section open science statement offers a reference link for the OSF datafiles. Please also provide a reference link for the MedRxiv preprint.

- We have added a reference for the MedRxiv preprint.

 

1. The first paragraph of the introduction includes two parenthetical references to https://asapbio.org. With another instance in the third paragraph of the discussion. Additional context clues this reference in the discussion indicate that the proper citation should be made the ASAPbio blog page from 2020-07-27 available here: https://asapbio.org/biopreprints2020-survey-initial-results. All ASAPbio citations should be updated to formally appear in the reference list with their direct blog links. The references in the introduction do not offer enough information to track down the intended citations but should be updated to the appropriate reference information as well. A fourth orphaned ASAPbio reference is also made in the second paragraph of the “External Factors…” section of the discussion.

- Following the F1000Research Guidelines for Research articles which states that, “Web links should be included as hyperlinks within the main body of the article, and not as references”, citations and references have been updated. We kindly thank you for your feedback. 

 

1. Final sentence of the Methods paragraph on Participant Recruitment predicts a 10% response rate. Since this is remarkably different the that 3.2% response rate, I wonder if the authors could provide any justification or rationale for the 10% prediction. Was this estimate based on similar author-response studies? Length/survey presentation/etc. Citations supporting this estimate would be appreciated for further research and study design.

- We conducted a survey involving individuals who were unfamiliar to us, anticipating a low response rate due to the challenges associated with recruiting participants for cross-sectional online surveys. In addition, we cannot tell how many authors had authors moved institutions, were on vacation, were ill, and passed away and thus, were unable to respond. We did try to increase the response rate by sending personalized reminders and keeping the survey completable in under 20 minutes long, as suggested by Menon and Muraleedharan (doi: 10.1136/gpsych-2020-100264).

 

1. I was confused by the inclusion of ResearchGate as a common preprint server to view/download preprints as presented in the Discussion section on Viewing/downloading preprints. Did 20.53% of respondents think downloading articles from ResearchGate equated to engaging with preprints? What about those cases where ResearchGate allows for downloading of the final publisher version? Can additional context around this survey question be provided to indicate how respondents thought ResearchGate related to preprints? I think to present it primarily as preprint server is a very concerning misunderstanding of the ResearchGate platform.

- We had listed ResearchGate as it is acknowledged as a preprint server on the ASAPBio preprint server directory ( https://asapbio.org/preprint-servers). We did not collect any specific information regarding respondents’ opinions of ResearchGate and whether this equated to engaging with preprinting.

 

1. I was startled by the data presented in Table 2 and I do not think the introduction appropriately framed the issue of mandating peer review in the preprinting process as something that would even be asked in such a survey. When the first sentence of the manuscript offers a definition of preprint as something “which has not yet undergone formal peer review” the survey question of mandating peer-review become incongruous with the definition of what is being studied. Similarly, if authors wish to present trust in peer-review as a key takeaway of the survey results, concerns around peer-review, and the role of preprints as one possible alternative, would be better introduced in the introduction of the manuscript.

We have described the issues around peer review and preprints as an alternative in the Background of the manuscript.

  

1. Table 3 respondent column should be presented as a percentage of the total responses for that question, as provided in all other figures.

- We have updated Table 3 to include the percentage as suggested.

 

1. The first paragraph of the discussion presents three main findings of the study that fear of misinformation, lack of peer review, and unsupportive journal policies were the most impactful preventative factors against preprinting. I am not convinced this is an appropriate representation of the data. While the first two points may indeed be primary concerns, I think unsupportive journal policies is an overstatement of the presented data which instead highlights respondents lack of awareness around journal policies and policy tools. While Figure 1 does show Journal Policy to be a more significant influence on choose to preprint than other factors, Figure 2 highlights that over 50% of authors aren’t familiar with journal policies at all or don’t remember the journal policies towards preprints for their last submissions. These data seem to support a lack of awareness on the part of the author, and not unsupportive journals. In fact, only 5% of authors are shown to be aware that their last journal for submission prohibited preprinting. The lack of author awareness is further discussed in the seventh paragraph of the discussion but the summarizing of this as “unsupportive journal policies” needs further justification. Relatedly, please add reference(s) to the claims that “most, but not all, life science journals accept preprinted manuscripts for submission” as this should be a key point of context. 

We have used the reviewer’s suggestions to put more emphasis of the “unfamiliarity of journal’s preprint policies” over “unsupportive journal policies”. We should note that there was a reference to the claim ““most, but not all, life science journals accept preprinted manuscripts for submission”.

# Reviewer report: Reviewer response for version 1

**DOI:** 10.5256/f1000research.156628.r280674

**Published:** 2024-06-08

**Authors:** Artemis Chaleplioglou

**Affiliations:** 1 Archival, Library & Information Studies (ALIS), University of West Attica, Aigaleo, Greece

This pre-registered cross-sectional online survey study ( https://doi.org/10.17605/OSF.IO/QA9GN) aims to assess biomedical researchers' attitudes towards preprinting as well as the factors, incentives and motivations that affect their opinions or decisions about preprints. Factors such as scientific journal policies, co-author consensus, lead author consensus, funding agency policies, and employers or host research institute perspectives were considered. The scientific questions posed by the authors are important in understanding the behavior of biomedical scientists in authoring, searching, accessing, reading, reviewing, citing, accepting or rejecting the evidence presented in preprints. The survey includes demographics, scientific publishing, preprint and peer review related questions. The collected survey data suggested a significant degree of familiarity with the preprint concept in biomedical scientific communication, viewing or downloading a preprint. However, the participants exhibited limited previous preprint contributions and lack of willingness to contribute or cite one in the future. Also, only a handful of the responders have participated in peer reviewing of preprints.

 The authors followed the sampling framework that was used by Ebrahimzadeh *et al* ( https://doi.org/10.17605/OSF.IO/8NKZF) in a similar contacted cross-sectional survey of biomedical researchers’ perceptions of reproducibility. Briefly, in the first step, the authors used the tens of thousands of journals of MEDLINE database to pick at random 400 out of them (approximately 1.3%). Then, in the second step the authors extracted the names and emails of paper contributors in these randomly selected journals between 2021/01/01 and 2022/08/01. Tens of thousands of authors were invited to participate and complete the survey.

 By default, the sampling framework design targeted predominantly to lead author investigators. This is because it is common for scientific journals to include only the correspondent author email addresses. Therefore, it is not surprising that most participants are academic faculty members (almost 80%). This is a strong point of the survey because the lead authors take the final decisions over the publishing or preprinting options for a paper. This finding was outlined in the report as the predominant limiting factor in preprint contributions.

 The fear of scooping may be among the factors and attitudes that strongly affect preprinting. There are investigators that because of this are hesitant over conference proceeding contributions as well.

 The predominant concern is about the first step of the random selection of journals from MEDLINE database (ncbijournals[All Fields]). Only a fraction of 12.5% of this list represents PubMed indexed journals (currentlyindexed[All Fields]). On top, in MEDLINE database catalog in addition to scientific journals there are other types of serial publications as well such as conference proceedings or workshops. It is not clear whether an additional screening was performed to ensure that only journals were included in the study. The odds of randomly picking a PubMed, Web of Science or SCImago indexed journal from this catalog appear to be pretty low. The odds may be even lower to pick high impact journals from the list. For this reason, it will add significantly if the authors provide the list of the journals or serial publications used in the survey as a supplement. This is also important because some journals actively support the submission to preprint servers whilst they implement preprint scooping-protection policies ( https://doi.org/10.1371/journal.pbio.3002502). The attitudes of the responders over preprints could be affected by the journals they published. Besides, the presentation of the randomly selected journals is important to ensure reproducibility.

 It could be postulated that clinical and pre-clinical researchers dominate the sample over basic research biomedical investigators because of the MEDLINE database content and the selection process. The Strengths and limitations section could be updated to include these limitations.

 In future studies it would be interesting to assess the attitudes of biomedical researchers preprint contributors over preprinting, open science, publishing, and traditional journal peer reviewing. A very interesting finding of this report is that despite the attitudes over preprinting almost 10% of the participants in the survey suggested that the peer review is bypassed.

Is the work clearly and accurately presented and does it cite the current literature?

Yes

If applicable, is the statistical analysis and its interpretation appropriate?

Yes

Are all the source data underlying the results available to ensure full reproducibility?

Partly

Is the study design appropriate and is the work technically sound?

Partly

Are the conclusions drawn adequately supported by the results?

Yes

Are sufficient details of methods and analysis provided to allow replication by others?

Partly

Reviewer Expertise:

Library and information science (LIS), Bibliometrics, Health information

I confirm that I have read this submission and believe that I have an appropriate level of expertise to confirm that it is of an acceptable scientific standard, however I have significant reservations, as outlined above.

## References

[1] : Recommendations for accelerating open preprint peer review to improve the culture of science. *PLoS Biol* .2024;22(2) : 10.1371/journal.pbio.3002502 e3002502 10.1371/journal.pbio.3002502 38421949 PMC10903809

# Response

**Published:** 2024-10-15

**Authors:** Jeremy Ng

**Affiliations:** University of Ottawa, Canada

**Reviewer 1**

**Comment: **This pre-registered cross-sectional online survey study ( https://doi.org/10.17605/OSF.IO/QA9GN) aims to assess biomedical researchers' attitudes towards preprinting as well as the factors, incentives and motivations that affect their opinions or decisions about preprints. Factors such as scientific journal policies, co-author consensus, lead author consensus, funding agency policies, and employers or host research institute perspectives were considered. The scientific questions posed by the authors are important in understanding the behavior of biomedical scientists in authoring, searching, accessing, reading, reviewing, citing, accepting or rejecting the evidence presented in preprints. The survey includes demographics, scientific publishing, preprint and peer review related questions. The collected survey data suggested a significant degree of familiarity with the preprint concept in biomedical scientific communication, viewing or downloading a preprint. However, the participants exhibited limited previous preprint contributions and lack of willingness to contribute or cite one in the future. Also, only a handful of the responders have participated in peer reviewing of preprints.

 The authors followed the sampling framework that was used by Ebrahimzadeh *et al*( https://doi.org/10.17605/OSF.IO/8NKZF) in a similar contacted cross-sectional survey of biomedical researchers’ perceptions of reproducibility. Briefly, in the first step, the authors used the tens of thousands of journals of MEDLINE database to pick at random 400 out of them (approximately 1.3%). Then, in the second step the authors extracted the names and emails of paper contributors in these randomly selected journals between 2021/01/01 and 2022/08/01. Tens of thousands of authors were invited to participate and complete the survey.

 By default, the sampling framework design targeted predominantly to lead author investigators. This is because it is common for scientific journals to include only the correspondent author email addresses. Therefore, it is not surprising that most participants are academic faculty members (almost 80%). This is a strong point of the survey because the lead authors take the final decisions over the publishing or preprinting options for a paper. This finding was outlined in the report as the predominant limiting factor in preprint contributions.

 The fear of scooping may be among the factors and attitudes that strongly affect preprinting. There are investigators that because of this are hesitant over conference proceeding contributions as well.

**Response: **We kindly thank you for providing your feedback on our manuscript.

**Comment: **The predominant concern is about the first step of the random selection of journals from MEDLINE database (ncbijournals[All Fields]). Only a fraction of 12.5% of this list represents PubMed indexed journals (currentlyindexed[All Fields]). On top, in MEDLINE database catalog in addition to scientific journals there are other types of serial publications as well such as conference proceedings or workshops. It is not clear whether an additional screening was performed to ensure that only journals were included in the study. The odds of randomly picking a PubMed, Web of Science or SCImago indexed journal from this catalog appear to be pretty low. The odds may be even lower to pick high impact journals from the list. For this reason, it will add significantly if the authors provide the list of the journals or serial publications used in the survey as a supplement. This is also important because some journals actively support the submission to preprint servers whilst they implement preprint scooping-protection policies ( https://doi.org/10.1371/journal.pbio.3002502). The attitudes of the responders over preprints could be affected by the journals they published. Besides, the presentation of the randomly selected journals is important to ensure reproducibility.

**Response: **We used specific filters from the NLM catalog to ensure that only journal entries were shown. This has been addressed in the Methods of the manuscript. A list of the 400 journals used were included in the Extended Data.

**Comment: **It could be postulated that clinical and pre-clinical researchers dominate the sample over basic research biomedical investigators because of the MEDLINE database content and the selection process. The Strengths and limitations section could be updated to include these limitations.

**Response: **The reviewer brings up a valid point, which we have now written about in the Strength and Limitations of the manuscript. 

**Comment: **In future studies it would be interesting to assess the attitudes of biomedical researchers preprint contributors over preprinting, open science, publishing, and traditional journal peer reviewing. A very interesting finding of this report is that despite the attitudes over preprinting almost 10% of the participants in the survey suggested that the peer review is bypassed.

**Response: **We agree with this suggestion and have briefly described this within the Discussion of the manuscript.
